# Key indicators for prioritizing swallowing assessment in acute ischemic stroke patients in the emergency room

**DOI:** 10.1055/s-0045-1812891

**Published:** 2025-11-21

**Authors:** Karoline Kussik de Almeida Leite, Fernanda Chiarion Sassi, Ana Paula Ritto, Claudia Regina Furquim de Andrade

**Affiliations:** 1 Universidade de São Paulo, Faculdade de Medicina, Hospital das Clínicas, Divisão de Miologia Oral, São Paulo SP, Brazil.; 2 Universidade de São Paulo, Faculdade de Medicina, Hospital das Clínicas, Departamento de Fisioterapia, Fonoaudiologia e Terapia Ocupacional, São Paulo SP, Brazil.

**Keywords:** Dysphagia, Stroke, Deglutition, Suction, Neurology

## Abstract

**Background:**

Stroke is a major cause of mortality and disability globally. Dysphagia is a frequent complication that increases the risk of aspiration pneumonia, a key contributor to stroke-related deaths. Early screening is essential for improving outcomes.

**Objective:**

To identify clinical indicators that can help prioritize swallowing assessments in the emergency room, enabling faster and safer resumption of oral feeding.

**Methods:**

A prospective cohort of 134 postacute ischemic stroke patients admitted to the emergency room was assessed. Patients were divided into 2 groups: G1 (at risk of dysphagia) and G2 (no risk). Swallowing function was evaluated using the Dysphagia Risk Evaluation Protocol (DREP) and the American Speech-Language-Hearing Association National Outcomes Measurement System (ASHA-NOMS) scale. A subset (n = 15) underwent videofluoroscopic swallowing study (VFSS). Stroke severity was measured using the National Institutes of Health (NIH) stroke scale (NIHSS). Statistical analyses included t-tests, Chi-squared test, Pearson's correlation, and Cochran's Q test ( *p* < 0.05).

**Results:**

Patients from G1 were older (mean: 69.1 vs. 63.0 years, *p* = 0.023), had more severe strokes (NIHSS ≥ 9.8, *p* = 0.002), and were more likely to require alternative feeding methods. Older age and longer hospital stays correlated with increased dysphagia risk. Coughing during the 50-ml water swallow test was a strong predictor of aspiration.

**Conclusion:**

Key indicators of aspiration risk in postacute ischemic stroke patients include age ≥ 69, NIHSS score ≥ 9, and the need for alternative feeding. Coughing during the water swallow test is a valuable clinical predictor. Early identification can support targeted interventions and reduce complications.

## INTRODUCTION

Stroke is the second leading cause of mortality and disability worldwide, and in Brazil, it is the leading cause of death and disability, making it one of the most significant public health challenges in the country. 1 2 In Brazil, between January and November 2024, around 177,590 individuals were hospitalized due to stroke, with 25,207 recorded deaths, reinforcing its position as the primary cause of mortality and disability in the country. 3 4

Advanced age is strongly associated with poorer functional recovery after ischemic stroke, including greater impairment in swallowing, mobility, and independence in activities of daily living. Studies consistently show that older patients, particularly those aged 80-years or older, have higher rates of functional dependence following stroke, regardless of the initial severity.

Saposnik et al. 5 demonstrated that age is an independent predictor of worse functional outcomes, with a higher proportion of elderly patients presenting moderate to severe disability both at hospital discharge and during long-term follow-up. Similarly, Smith et al. 6 reported that patients aged ≥ 80 undergoing intra-arterial therapy had significantly worse functional outcomes, with a lower likelihood of achieving functional independence at 90 days compared to younger patients. These results emphasize that beyond the risk of clinical complications, aging has a direct impact on functional recovery, including swallowing abilities, underscoring the importance of early assessment and intervention in elderly, poststroke patients.

Dysphagia is a prevalent complication of stroke globally, affecting almost 50% of patients. Those with this condition face a significantly higher risk of aspiration pneumonia, which is a leading cause of death among stroke patients. Early screening for dysphagia has been shown to decrease the incidence of aspiration pneumonia, thereby enhancing nutritional support and mitigating risks. 7

The American Heart Association/American Stroke Association indicates that patients who fail dysphagia screening require twice the hospitalization time and are up to seven times more likely to develop pneumonia compared to those who pass the screening. Therefore, early speech therapy screening is crucial within the first hours of hospitalization for all stroke patients, preceding the recommendation and prescription of oral diets and medications. Early management of these patients aims to minimize the rate of hospital-related clinical complications. 8 9

In Brazil, the 2021 Clinical Protocol and Therapeutic Guidelines for Acute Ischemic Stroke 10 included dysphagia screening as part of the initial assessment before initiating oral feeding. However, despite recognizing the importance of early detection, the protocol remains superficial in its recommendations for dysphagia management. It fails to provide clear criteria for conducting clinical evaluations, lacks guidance on which validated screening tools should be used, and omits recommendations for instrumental assessments, such as videofluoroscopy or fiberoptic endoscopic evaluation, even in high-risk cases.

Additionally, the protocol does not offer detailed pathways for clinical decision-making regarding diet consistency, compensatory strategies, or oral feeding progression. This limited approach may contribute to inconsistent practices across hospitals and does not adequately address the complexity of managing dysphagia in poststroke patients, particularly considering the significant functional impact swallowing disorders have on recovery. Therefore, although it represents progress compared to previous manuals, the current guideline still falls short of meeting the clinical demands for comprehensive and safe dysphagia care.

Dysphagia screening has beneficial effects for stroke patients. The multidisciplinary team in stroke units and rehabilitation facilities plays a crucial role in implementing screening procedures. Various healthcare professionals can receive training in dysphagia identification and should actively participate in the screening process. 11

In Brazil, stroke patients typically spend at least 48 hours in emergency units receiving initial care, necessitating prompt and well-founded medical decisions. However, there are no current updated protocols tailored to the Brazilian population to guide healthcare teams in safely introducing oral diets. This gap can prolong hospital stays, increase service costs, and raise the risk of hospital-acquired infections.

The aim of this study was to develop a dysphagia screening protocol for acute ischemic stroke patients to be used in the emergency room (ER) by a medical and multidisciplinary team, based on clinical predictors of aspiration risk identified by speech-language pathologists.

## METHODS

We conducted a prospective observational cohort study of patients with dysphagia following acute ischemic stroke who were admitted to the ER. The study protocol was approved by the Scientific and Ethics Committee of the Hospital das Clínicas of the Faculdade de Medicina from the Universidade de São Paulo (HCFMUSP), under the number 3.691.262. Informed consent was waived as the study relied on analysis of medical records.

### Patient population

Patients were eligible for inclusion if they met the following criteria: admission to the ER from May 2022 to 2023; diagnosis of acute ischemic stroke confirmed by neurological evaluation and computed tomography (CT) scan; bedside swallowing evaluation (BSE) and swallowing treatment requested by the primary treating physician, performed by a speech-language pathologist; age ≥ 18 years; clinical and respiratory stability; Glasgow coma scale score ≥ 13 points; no history of feeding complaints or dietary changes; no previous use of alternative feeding methods; no tracheostomy; and no history of head and neck surgery. The participants were selected using a convenience sampling method, which included all consecutive patients who met the eligibility criteria during the data collection period.

According to the literature, the risk of death can increase up to three times for patients who develop aspiration pneumonia during hospitalization, 12 13 highlighting the importance of early dysphagia assessment in poststroke patients to minimize clinical complications. For these reasons, the protocol adopted for this study mandated that acute phase stroke patients be assessed within 48 hours of the ischemic stroke onset (ictus). Due to limitations such as clinical condition, patient positioning, displacement, and high costs, it was not possible to conduct the gold standard test for aspiration (videofluoroscopy of swallowing) for every case. Out of the 134 patients evaluated, 15 were randomly selected for objective swallowing assessment.

### Clinical assessment of swallowing

The clinical evaluation of swallowing was performed by speech-language pathologists (SLPs) from the Division of Oral Myology of the same institution within 48 hours of hospital admission. The assessment followed the Dysphagia Risk Evaluation Protocol, Screening Version (DREPs). 14 This is a Brazilian protocol, published and validated in 2020, designed for early bedside screening of penetration and aspiration risk. Its application involves the administration of controlled volumes of water and puree to assess the patient's ability to swallow safely.

The DREPs has been validated, showing a sensitivity of 92.9%, specificity of 75.0%, positive predictive value (PPV) of 65.0%, negative predictive value (NPV) of 95.5%, and accuracy of 80.9%. 14 It is important to note that the protocol adopts a sequential progression model, meaning that larger volumes are only tested if the patient safely completes the smaller volumes. Therefore, if a patient exhibits clinical signs of dysphagia (e.g., coughing, wet voice, or choking) at a smaller volume, the test is interrupted, and larger volumes are not administered. This criterion, while essential for patient safety, limits the ability to analyze the distribution of clinical signs across all volume levels, as not all participants were exposed to the full range of tested volumes, including the largest (50 mL). This methodological characteristic should be considered when interpreting volume-based analyses of swallowing performance.

Swallowing functionality was classified using the American Speech-Language-Hearing Association National Outcomes Measurement System (ASHA NOMS) scale, 15 with a range from levels 1 (unable to swallow safely) to 7 (feeds independently with no limitations).

The swallowing functional level of each patient was classified as follows:

- Level 1: The individual cannot swallow anything safely through the mouth, and all nutrition and hydration are administered via nonoral routes.
- Level 2: The individual cannot swallow safely through the mouth but can ingest some items of various consistencies only during therapy sessions, and an alternative feeding route is required.
- Level 3: An alternative feeding route is needed, as the individual ingests less than 50% of their nutrition and hydration orally, or swallowing is safe with moderate use of compensatory strategies.
- Level 4: Swallowing is safe but often requires moderate use of compensatory strategies; the individual may still need feeding by an alternative route or oral supplementation.
- Level 5: Swallowing is safe with minimal dietary restrictions; the individual occasionally requires minimal clues for compensatory strategies and can self-monitor, with all nutrition and hydration received orally during meals.
- Level 6: Swallowing is safe, and the individual eats and drinks independently, rarely needing compensatory strategies and often self-monitoring, although specific foods may need to be avoided.
- Level 7: The individual feeds independently, with no limitations on swallowing function; feeding is safe and efficient for all consistencies, and compensatory strategies are effectively used when necessary.

To ensure reliability, all speech therapists responsible for evaluating bedside received specific training to assess functional level during the first clinical evaluation and upon resolution of dysphagia or at hospital discharge. The evaluations were conducted by speech therapists with experience in treating dysphagia, all trained to apply the same treatment protocol.

### Objective evaluation of swallowing – videofluoroscopic swallowing study (VFSS)

Following the DREP application, one in every 9 patients was randomly selected for videofluoroscopic swallowing study (VFSS) within 24 hours. A total of 15 patients underwent the exam at the Radiology Institute of Hospital das Clínicas, University of São Paulo (InRad/HCFMUSP), conducted by a radiologist and a speech therapist, both blinded to the clinical assessment. The fluoroscopy equipment used was the GE Medical Systems ADVANTX (GE Healthcare). Patients were assessed in seated position (90°) from lateral and anteroposterior views during ingestion of barium-diluted liquids (3, 5, 10, and 50 ml). The VFSS images were recorded and analyzed using VirtualDub software (Open Source). Swallowing impairment was classified using the Rosenbek scale, 16 with dysphagia defined as a score ≥ 3.

### Severity of stroke

The severity of stroke was assessed using the National Institutes of Health stroke scale (NIHSS) 17 calculated at the time of ER admission by a neurologist.

### Data analysis

Data were statistically analyzed using the IBM SPSS Statistics for Windows (IBM Corp.), version 25. Quantitative data were described with mean and standard deviation (SD), and inferential analysis was conducted to compare groups using Student's t-test. Qualitative data were analyzed descriptively (total count and percentage) and inferentially using Pearson's Chi-squared test. A correlation analysis was performed using Pearson's correlation coefficient. Intragroup differences in liquid volume failure during DREP were assessed using Cochran's Q test to evaluate the association between volume levels (3, 5, 10, and 50 ml) and failure rates. The post hoc analyses identified specific volume comparisons contributing to observed differences. Statistical significance was determined as *p* -value < 0.05.

### Data availability

The underlying data supporting this study consists of sensitive health information that cannot be publicly shared due to ethical and legal restrictions. However, the data will be made available upon reasonable request to the corresponding author after publication, provided that the requester has appropriate institutional approvals and complies with data protection regulations.

## RESULTS

The 134 participants in this study were divided into two groups based on their DREP results. Participants who failed at least one of the clinical predictors (cough, choking, wet voice, altered cervical auscultation) during the water swallow test were grouped into G1–at risk for dysphagia (n = 42). Participants who did not fail any of these items were grouped into G2–no risk for dysphagia (n = 92). There was no difference between the groups regarding stroke lateralization (i.e., similar distribution of right and left hemisphere involvement) or vascular territory (i.e., 86.79% carotid, and 13.20% vertebrobasilar involvement). The groups were compared according to their demographic variables, as presented in Table 1 . The results indicated significant differences between the groups for the variable age, with G1 showing a higher age compared to G2.

**Table 1:** Between-group comparisons for demographic and clinical characteristics

		G1 (n = 42)	G2 (n = 92)	*p* -value
Age in years	Mean ± SD	69.1 ± 16.0	63.0 ± 13.3	0.023*
Sex, n (%)	Male	24 (57.1%)	62 (67.4%)	0.254
	Female	18 (42.9%)	30 (32.6%)	
Time between speech therapy evaluation and hospital discharge (days)				
Mean ± SD		11.7 ± 12.4	2.7 ± 2.7	< 0.001*
Participants recommended alternative feeding methods following speech therapy evaluation				
N (%)		18 (42.9%)	5 (5.4%)	0.275

Abbreviations: DREP, Dysphagia Risk Evaluation Protocol; G1, at risk for dysphagia, participants who failed DREP; G2, no risk for dysphagia, participants who passed DREP; n, number of participants; SD, standard deviation.

Note: Significant difference according to Student's t-test.

The number of participants who failed the clinical predictors in the DREP (cough, choking, wet voice, and altered cervical auscultation) was compared between groups in Table 2 . It is important to note that participants who failed at 3-, 5-, 10-, and 50-ml liquid volumes were not tested at subsequent volumes.

**Table 2:** G1 intragroup comparison of the liquid volume at which DREP failure occurred

Liquid volume in DREP		Participants n (%)	*p* -value
Cough	3 ml	9 (21.4%)	0.024*
	5 ml	6 (14.3%)	
	10 ml	3 (7.1%)	
	50 ml	16 (38.1%)	
	None	8 (19.0%)	
Choking	3 ml	6 (14.3%)	< 0.001*
	5 ml	4 (9.5%)	
	10 ml	1 (2.4%)	
	50 ml	5 (11.9%)	
	None	26 (61.9%)	
Altered cervical auscultation	3 ml	3 (7.1%)	< 0.001*
	5 ml	1 (2.4%)	
	10 ml	2 (4.8%)	
	50 ml	1 (2.4%)	
		35 (83.3%)	
Wet voice	3 ml	2 (4.8%)	< 0.001*
	5 ml	0 (0%)	
	10 ml	0 (0%)	
	50 ml	1 (2.4%)	
	None	39 (92.9%)	

Abbreviations: DREP, Dysphagia Risk Evaluation Protocol; G1, at risk for dysphagia, participants who failed DREP.

Note: *significant difference according to Cochran's Q test for Related Samples. The DREP protocol follows a progressive volume testing procedure in which larger volumes are only tested if the patient safely swallows smaller volumes. Therefore, the number of participants tested at higher volumes (e.g., 50 mL) is lower, as testing is interrupted in cases of failure at smaller volumes. This should be considered when interpreting the distribution of clinical signs across different volumes.

Table 2 presents the results of the intragroup comparison within G1 of the liquid volume at which DREP failure occurred. For the clinical sign “cough,” post hoc pairwise comparison with Bonferroni correction indicated a significant difference only between the test conducted with 10 and 50 ml ( *p* = 0.014). For the clinical signs “choking,” “altered cervical auscultation,” and “wet voice,” post hoc pairwise comparison with Bonferroni correction indicated a significantly higher number of participants who did not present this clinical sign in DREP, compared to those who did ( *p* < 0.001 for all comparisons).

Table 3 , which shows the comparison of the groups regarding NIHSS results related to stroke severity, indicated significant differences for mild severity: there was a higher proportion of participants with mild stroke in G2 ( *p* = 0.018) according to Pearson's Chi-squared test. However, G1 had a higher proportion of participants classified as severe.

**Table 3:** Intergroup comparison regarding neurological dysfunction, according to the NIHSS

	G1 (n = 42)	G2 (n = 92)	*p* -value
NIHSS (mean ± SD)	9.8 ± 5.9	6.5 ± 4.7	0.002*
Mild	24 (57.1%)	71 (77.2%)	0.018**
Moderate	8 (19.0%)	11 (12.0%)	0.275
Severe	10 (23.8%)	10 (10.9%)	0.051

Abbreviations: DREP, Dysphagia Risk Evaluation Protocol; G1, at risk for dysphagia, participants who failed DREP; G2, no risk, participants who passed DREP; n, number of participants; NIHSS, National Institute of Health Stroke Scale; SD, standard deviation; %, percentage of participants.

Notes: *significant difference according to Student's t-test; **significant difference according to Pearson's chi-square test.

A correlation analysis was conducted among the variables age, gender, time between speech therapy evaluation and hospital discharge, and number of participants who were recommended alternative feeding methods following speech therapy evaluation. According to the data presented in Table 4 , the DREP results showed significant positive correlation with the variables age, time between speech therapy evaluation and hospital discharge, and participants recommended alternative feeding methods following speech therapy evaluation. However, the degree of association for each variable differed.

**Table 4:** Correlation of demographic variables and medical record data with the results of the DREP

Variables	r	*p-* value
Age	0.196	0.023*
Gender	−0.099	0.254
Time between speech therapy evaluation and hospital discharge (days)	0.446	< 0.001*
Participants who were recommended alternative feeding methods following speech therapy evaluation	0.384	< 0.001*

**Abbreviation** : DREP, Dysphagia Risk Evaluation Protocol.

**Notes:** 0 < r < 0.25, low or no association; 0.25 < r < 0.5, weak degree of association; 0.5 < r < 0.75, moderate or strong degree of association; r > 0.75, strong or excellent degree of association; * significant difference according to Pearson's correlation coefficient.

**Figure 1:**
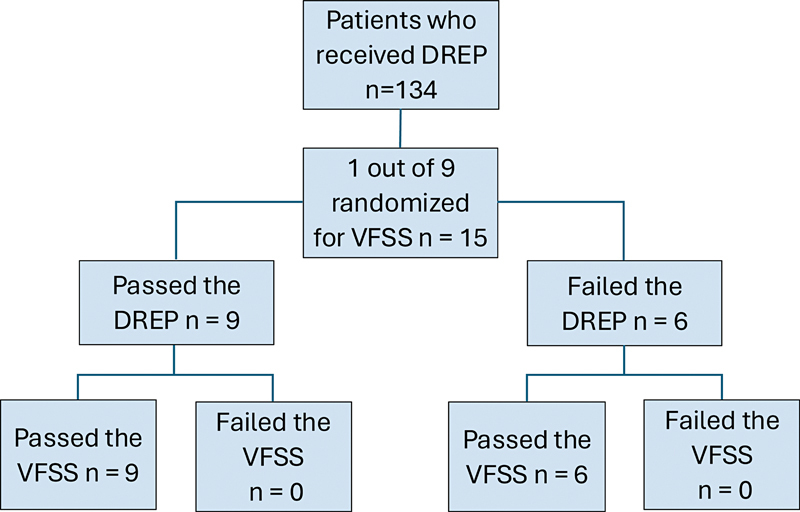
Abbreviations: n, number of subjects; DREP, dysphagia risk assessment protocol; VFSS, videofluoroscopic swallowing study. Selection of patients for VFSS.

Among the 134 patients evaluated, a random sample of 15 was selected for the objective swallowing assessment. Of the 15 patients who underwent the VFSS, only 6 failed the clinical evaluation of the DREP, exhibiting coughing, and none failed the objective assessment. Figure 1 illustrates the flow diagram for the selection of patients who underwent the VFSS.

## DISCUSSION

The present study examined risk indicators for aspiration pneumonia in postacute ischemic stroke patients admitted to the ER of HCFM-USP. So far, this is one of the few Brazilian studies assessing potential predictors of aspiration pneumonia using clinical indicators in this population. Establishing prioritization indicators for speech therapy in patients with acute ischemic stroke is crucial for reducing hospital costs, optimizing bedside speech evaluations, and ensuring early and safe return to oral feeding through dysphagia screening.

In general, the results of the present study indicated that patients at risk of aspiration pneumonia had an average age exceeding 69 years, greater neurological impairment according to the NIHSS score, and a higher indication for alternative feeding methods following speech therapy evaluation. The clinical predictor of aspiration pneumonia that most differentiated the groups was the presence of cough at the 50 ml volume.

The literature 1 18 indicates that age has a significantly greater impact on the occurrence of poststroke dysphagia in the elderly population, supporting the findings of this study. Its prevalence can be 20 to 40% higher for individuals over 55-years-old compared to the general population (6–9%). 14 19 Normal aging is typically associated with cerebral atrophy, neural function decline, and reduced muscle mass, 16 which may influence the swallowing process.

The literature also points to the association between age and decreased activation of airway protection mechanisms during swallowing, favoring bronchopulmonary penetration/aspiration. 19 20 These factors could justify the higher occurrence of postswallowing cough observed in this study, especially among patients aged over 69 years.

In the present study, patients at risk of aspiration not only exhibited older age but also had more severe stroke grades, according to the NIHSS. While this scale does not directly assess swallowing, it includes parameters that aid in the functional clinical evaluation of the patient, such as level of consciousness, facial paralysis, language disorders, dysarthria, and motor and sensory deficits. 18 Therefore, although the NIHSS is not a validated measure for dysphagia screening, it has been widely used in many studies as a complementary tool for swallowing assessment, showing a positive correlation with the presence of dysphagia and pneumonia. 21 22 23 24

The literature has already identified that the cognitive, physical, and sensory deficits observed in poststroke individuals often result in swallowing disorders, which increase morbidity and may lead to premature death. 25 Moreover, a reduced level of consciousness impacts the swallowing mechanism, potentially leading to aspiration. 25 26 27 A study correlated the risk of aspiration and stroke severity using the NIHSS and found that patients with more neurological deficits were more likely to develop pneumonia. 28 Another study, 29 with a sensitivity of 88% and a specificity of 85%, used the NIHSS as a risk selection criterion for dysphagia in the acute phase of stroke, developing an algorithm in which NIHSS < 10 allows for oral feeding and NIHSS ≥14 indicates the need for alternative feeding methods. The results of the present study identified NIHSS ≥ 9 as an individual predictor of dysphagia in patients with acute ischemic stroke.

Previous studies 8 13 15 have demonstrated that the need for alternative poststroke feeding methods correlates with a higher mortality rate and poorer outcomes, although the use of feeding tubes reduces the risk of bronchoaspiratory pneumonia. In the present study, over 40% of patients at risk of dysphagia were recommended alternative feeding methods after a speech-language pathology evaluation. Despite being at risk, this was not required for the others, as their oral diet could be adapted to a safer consistency, with the majority only needing thickening agents for liquids.

The present study identified the presence of coughing during the water test as a predictor of aspiration risk. Regarding dysphagia screening tests in patients in the acute phase of stroke, the literature predominantly presents protocols that utilize only water offering for swallowing assessments. 30 31 The water tests were the first described in the literature and are currently the most used in this population; however, studies differ regarding the offered volume. 30 31 32 One study conducted an objective swallowing assessment in individuals with acute stroke and observed that aspiration of liquids and silent aspiration are characteristics of dysphagia in these patients. Another 33 demonstrated that offering a larger volume of water (90 ml) stimulates the cough reflex, potentially preventing silent aspiration with smaller volumes. In this study, 81% of patients at risk of aspiration presented coughing during the clinical swallowing assessment, with most using 50 ml of water (47%).

Regarding the objective assessment, 20% of the patients who underwent VFSS exhibited the clinical sign of coughing both during the examination and in the clinical assessment (all presented coughing with 50 ml of water).Despite this, these patients were considered a failure in the DREP but were not classified as such in the VFSS, as none had a score greater than or equal to 3 on the Rosenbek scale, 16 indicating that there was no liquid aspiration in these patients. Another 20% presented with coughing only in the clinical assessment with the DREP and showed no clinical signs during the VFSS.

To confirm the specificity and sensitivity of the DREP in this population, future studies with a larger number of participants will be necessary. The lower prevalence of patients at risk for aspiration in the sample that underwent VFSS may be justified by their lower severity of the stroke and greater clinical stability, which were prerequisites for the possibility of transportation and conducting the examination.

This study has several limitations that warrant consideration. Firstly, the sample was drawn from a single institution, which may restrict the generalizability of the findings. Therefore, multicenter studies involving larger and more diverse populations are needed to validate these results across different clinical settings.

Secondly, there is an inherent selection bias in the subgroup undergoing VFSS, as only patients with lower stroke severity, greater clinical stability, and adequate conditions for safe transport and positioning were able to undergo the examination. This likely contributed to the lower prevalence of aspiration risk observed in this subgroup compared to the overall cohort.

Furthermore, although the DREP clinical screening tool demonstrated high sensitivity, it may yield false positives when compared to instrumental assessments, underscoring the importance of complementing bedside evaluations with objective methods, such as VFSS or fiberoptic endoscopic evaluation of swallowing (FEES), whenever feasible. Nevertheless, we acknowledge that in many acute care settings, logistical challenges (i.e. patient severity, limited mobility, and restricted access to specialized equipment) often hinder the routine use of such instrumental assessments.

Lastly, the integration of the NIHSS as a complementary tool for prioritizing speech-language pathology evaluations is a significant strength of this study. This approach holds promise for improving clinical decision-making and should be further investigated and validated in future research across diverse healthcare environments.

In conclusion, the primary objective proposed in this study was to provide indicators for prioritizing speech-language assessments, allowing for the identification of patients at higher risk of aspiration and directing them to appropriate procedures, ensuring a quicker and safer return to oral feeding, thus reducing the high incidence of morbidities and preventing pulmonary complications resulting from aspiration, enabling shorter hospitalization times.

Based on these findings, we propose a dysphagia screening protocol for patients with acute ischemic stroke in the emergency room. Those who meet the following criteria: age > 69 years, NIHSS score ≥ 9, and coughing after swallowing during the bedside water test (50 mL); should be considered at high risk for dysphagia. Among these predictors, the NIHSS score ≥ 9 stands out as a particularly strong and practical clinical indicator. This finding reinforces previous evidence linking neurological severity to swallowing dysfunction and highlights this scale's relevance as an accessible and reliable tool to support decision-making in emergency settings.

The patients must maintain fasting until a comprehensive speech-language pathology evaluation is performed. If necessary, confirmation of dysphagia should be obtained through imaging studies to guide appropriate management and prevent complications, such as aspiration pneumonia. This protocol aims to optimize the early identification of dysphagia in acute stroke patients, improving clinical outcomes, enhancing patient safety, and potentially reducing hospitalization time.
